# Supplementary material for: Rapid Detection of Carbapenemases Using NG-Test® CARBA 5 in Positive Blood Cultures: A Diagnostic Test Study
Source: Antibiotics (Basel). 2024 Nov 20;13(11):1105. doi: 10.3390/antibiotics13111105 (PMC11591157; doi:10.3390/antibiotics13111105)
Supplement: Supplementary file 1 [file antibiotics-13-01105-s001.zip › antibiotics-3286183-supplementary.pdf]

antibiotics

*SUPPLEMENTARY MATERIAL*

# Rapid detection of carbapenemases using NG-Test® CARBA 5 in positive blood cultures, a diagnostic test study

**Diana Munguia-Ramos [1], Luis Fernando Xancal-Salvador [2], Verónica Esteban-Kenel [2], Narciso Ortiz-Conchi [2], Ricardo Antonio Jaimes-Aquino [2], Miguel Mendoza-Rojas [2], Axel Cervantes-Sánchez [2], Steven Méndez-Ramos [2], Hector Orlando Rivera-Villegas [1], Sandra Rajme-Lopez [1], Karla Maria Tamez-Torres [1,2], Carla Marina Roman-Montes [1,2], Areli Martínez-Gamboa [2], Miriam Bobadilla del-Valle [2], Jose Sifuentes-Osornio [3], Alfredo Ponce-de-Leon [1], Maria Fernanda Gonzalez-Lara [1,2], Bernardo Alfonso Martinez-Guerra [1,2]***

[1] Instituto Nacional de Ciencias Médicas y Nutrición Salvador Zubirán, Department of Infectious Diseases, 15 Vasco de Quiroga, Belisario Domínguez Secc 16, Tlalpan, 14080 Mexico City, Mexico.

[2] Instituto Nacional de Ciencias Médicas y Nutrición Salvador Zubirán, Department of Infectious Diseases, Clinical Microbiology Laboratory, 15 Vasco de Quiroga, Belisario Domínguez Secc 16, Tlalpan, 14080 Mexico City, Mexico.

[3] Instituto Nacional de Ciencias Médicas y Nutrición Salvador Zubirán, General Direction, 15 Vasco de Quiroga, Belisario Domínguez Secc 16, Tlalpan, 14080 Mexico City, Mexico.Affiliation 1; e-mail@e-mail.com

* Correspondence: Martinez-Guerra Bernardo Alfonso. Telephone +52 55 54 87 09 00 ext. 5860. beramg@gmail.com, bernardo.martinezg@incmnsz.mx

**Table S1.** Complete antimicrobial susceptibility results and agreement

| Sample | Isolate | MIC CRO | MIC CAZ | MIC ETP | MIC MEM | MIC IPM | mCIM | eCIM | Carbapenemase-encoding genes | NG CARBA 5 | Agreement between mCIM and NG Test ® Carba 5 | Agreement between PCR and NG Test ® Carba 5 |
|---|---|---|---|---|---|---|---|---|---|---|---|---|
| 1 | *E. coli* | ≧64 /R | ≧64/R | 2/I | 1/S | 1/S | Negative | - | None detected | Negative | Yes | Yes |
| 2 | *E. coli* | ≧64/R | ≧64/R | 0.5/S | 1/S | 2/I | Negative | - | None detected | Negative | Yes | Yes |
| 3 | *E. coli* | ≧64/R | ≧64/R | ≧8/R | ≧32/R | ≧32/R | Positive | Positive | NDM | NDM | Yes | Yes |
| 4 | *E. coli* | ≧64/R | ≧64/R | ≧16/R | ≧32/R | ≧16/R | Positive | Positive | NDM | NDM | Yes | Yes |
| 5 | *E. coli* | ≧64/R | 16/R | 4/R | ≤1/S | 4/R | Positive | Negative | OXA-48 | OXA-48-Like | Yes | Yes |
| 6 | *E. coli* | ≧64/R | 16/R | 4/R | ≤1/S | 4/R | Positive | Negative | OXA-48/NDM | OXA-48-Like | No | No |
| 7 | *E. coli* | ≧64/R | ≧64/R | ≧8/R | >=32/R | ≧32/R | Positive | Positive | NDM | NDM | Yes | Yes |
| 8 | *E. coli* | ≧64/R | ≧64/R | ≧16/R | ≧32/R | ≧32/R | Positive | Positive | NDM | NDM | Yes | Yes |
| 9 | *E. coli* | ≧64/R | ≧64/R | ≧8/R | ≧32/R | ≧32/R | Positive | Negative | OXA-48/NDM | OXA-48-Like/NDM | No | Yes |
| 10 | *E. coli* | ≧16/R | 4/S | 2/R | 0.5/S | 1/S | Positive | Negative | OXA-48 | OXA-48-Like | Yes | Yes |
| 11 | *E. coli* | ≧64/R | ≧64/R | ≧8/R | ≧16/R | 8/R | Positive | Negative | OXA-48/NDM | OXA-48-Like/NDM | No | Yes |
| 12 | *K. pneumoniae* | ≧64/R | ≧64/R | ≧16/R | >=32/R | >=32/R | Positive | Negative | KPC/NDM | KPC/NDM | No | Yes |
| 13 | *K. pneumoniae* | ≧64/R | ≧64/R | ≧16/R | >=32/R | >=32/R | Positive | Negative | KPC/NDM | KPC/NDM | No | Yes |
| 14 | *K. pneumoniae* | ≧64/R | ≧64/R | ≧16/R | ≧32/R | ≧32/R | Positive | Negative | KPC/NDM | KPC/NDM | No | Yes |
| 15 | *K. pneumoniae* | ≤ 1/S | ≤1/S | ≤0.5/S | ≤0.25/S | ≤0.25/S | Negative | - | None detected | Negative | Yes | Yes |
| 16 | *K. pneumoniae* | ≧64/R | ≧64/R | ≧16/R | 2/I | 2/I | Positive | Positive | OXA-48 | OXA-48-Like | Yes | Yes |
| 17 | *E. cloacae* | ≧64/R | ≧64/R | 8/R | 2/I | 2/I | Negative | - | GES | Negative | Yes | Yes |
| 18 | *E. cloacae* | ≤1/S | ≤1/S | ≤0.5/S | ≤0.25/S | ≤0.25/S | Negative | - | None detected | Negative | Yes | Yes |
| 19 | *E. cloacae* | ≧64/S | ≧64/R | 16/R | 2/I | 4/R | Negative | - | None detected | Negative | Yes | Yes |
| 20 | *K. aerogenes* | ≧64/R | ≧64/R | 8/R | 1/S | 4/R | Negative | - | None detected | Negative | Yes | Yes |
| 21 | *R. ornithinolytica* | ≤1/R | ≤1/S | 4/R | 1/S | 2/I | Positive | Negative | OXA-48 | OXA-48-Like | Yes | Yes |
| 22 | *P. aeruginosa* | ≧64/R | ≧64/R | NA | ≧32/R | 16/R | Negative | - | GES | Negative | Yes | Yes |
| 23 | *P. aeruginosa* | ≧64/R | ≧64/R | NA | 4/I | ≧32/R | Negative | - | None detected | Negative | Yes | Yes |

| | | | | | | | | | | | | |
|---|---|---|---|---|---|---|---|---|---|---|---|---|
| 24 | *P. aeruginosa* | 32/R | 4/S | NA | 4/I | ≧32/R | Negative | - | None detected | Negative | Yes | Yes |
| 25 | *P. aeruginosa* | ≧64/R | 8/S | NA | 8/R | 16/R | Negative | - | None detected | Negative | Yes | Yes |
| 26 | *P. aeruginosa* | ≧64/R | ≧64/R | NA | ≧32/R | 32/R | Negative | - | None detected | Negative | Yes | Yes |
| 27 | *P. aeruginosa* | 32/R | ≤1/S | NA | 4/I | 16/R | Negative | - | None detected | Negative | Yes | Yes |
| 28 | *P. aeruginosa* | ≧64/R | ≤1/S | NA | 8/R | 16/R | Negative | - | None detected | Negative | Yes | Yes |
| 29 | *P. aeruginosa* | 16/R | 4/S | NA | 4/I | ≧32/R | Negative | - | None detected | Negative | Yes | Yes |
| 29 | *P. aeruginosa* | 64/R | ≧64/R | NA | 32/R | 32/R | Positive | Positive | VIM | VIM | Yes | Yes |
| 30 | *P. aeruginosa* | ≧64/R | 4/S | NA | 16/R | ≧32/R | Negative | - | None detected | Negative | Yes | Yes |
| 31 | *P. aeruginosa* | 32/R | 8/S | NA | 8/R | ≧32/R | Negative | - | None detected | Negative | Yes | Yes |

I: intermediate susceptibility, R: resistant, S: susceptible , CRO: ceftriaxone, CAZ: ceftazidime, ETP: ertapenem, MEM: meropenem, IPM : imipenem
